# Aeolian transport of viable microbial life across the Atacama Desert, Chile: Implications for Mars

**DOI:** 10.1038/s41598-019-47394-z

**Published:** 2019-08-22

**Authors:** Armando Azua-Bustos, Carlos González-Silva, Miguel Ángel Fernández-Martínez, Cristián Arenas-Fajardo, Ricardo Fonseca, F. Javier Martín-Torres, Maite Fernández-Sampedro, Alberto G. Fairén, María-Paz Zorzano

**Affiliations:** 1 Centro de Astrobiología (CSIC-INTA), 28850 Madrid, Spain; 2 Instituto de Ciencias Biomédicas, Facultad de Ciencias de la Salud, Universidad Autónoma de Chile, Santiago, Chile; 3 Facultad de Ciencias, Universidad de Tarapacá, Arica, Chile; 4 Atacama Biotech, Santiago, Chile; 5 Division of Space Technology, Department of Computer Science, Electrical and Space Engineering, Luleå University of Technology, Luleå, Sweden; 6 Instituto Andaluz de Ciencias de la Tierra (UGR-CSIC), Armilla, Granada, Spain; 7 Department of Astronomy, Cornell University, Ithaca, 14853 NY USA

**Keywords:** Air microbiology, Astrobiology

## Abstract

Here we inspect whether microbial life may disperse using dust transported by wind in the Atacama Desert in northern Chile, a well-known Mars analog model. By setting a simple experiment across the hyperarid core of the Atacama we found that a number of viable bacteria and fungi are in fact able to traverse the driest and most UV irradiated desert on Earth unscathed using wind-transported dust, particularly in the later afternoon hours. This finding suggests that microbial life on Mars, extant or past, may have similarly benefited from aeolian transport to move across the planet and find suitable habitats to thrive and evolve.

## Introduction

The Atacama is the driest and oldest Desert on Earth, and a well-known Mars analog environment^1–6^. The driest sites of the Atacama have all been reported in the central valley of this desert (known as the hyperarid core) (Fig. S1), characterized by extremely low precipitations, extremely high UV radiation and an atmospheric relative humidity that usually drop to zero in the afternoon hours^1,2,6^, with sites reported as dry as Mars^6^.

Despite these extreme conditions, microbial life in the hyperarid core have been reported even in its driest sites, where water activity in its soils get as low as 0.15^6^. Here we report that viable microbial life is indeed able to traverse the hyperarid core of the Atacama on dust transported by the wind, also suggesting the sources of origin of the species found in the hyperarid core of this desert.

**Figure 1:**
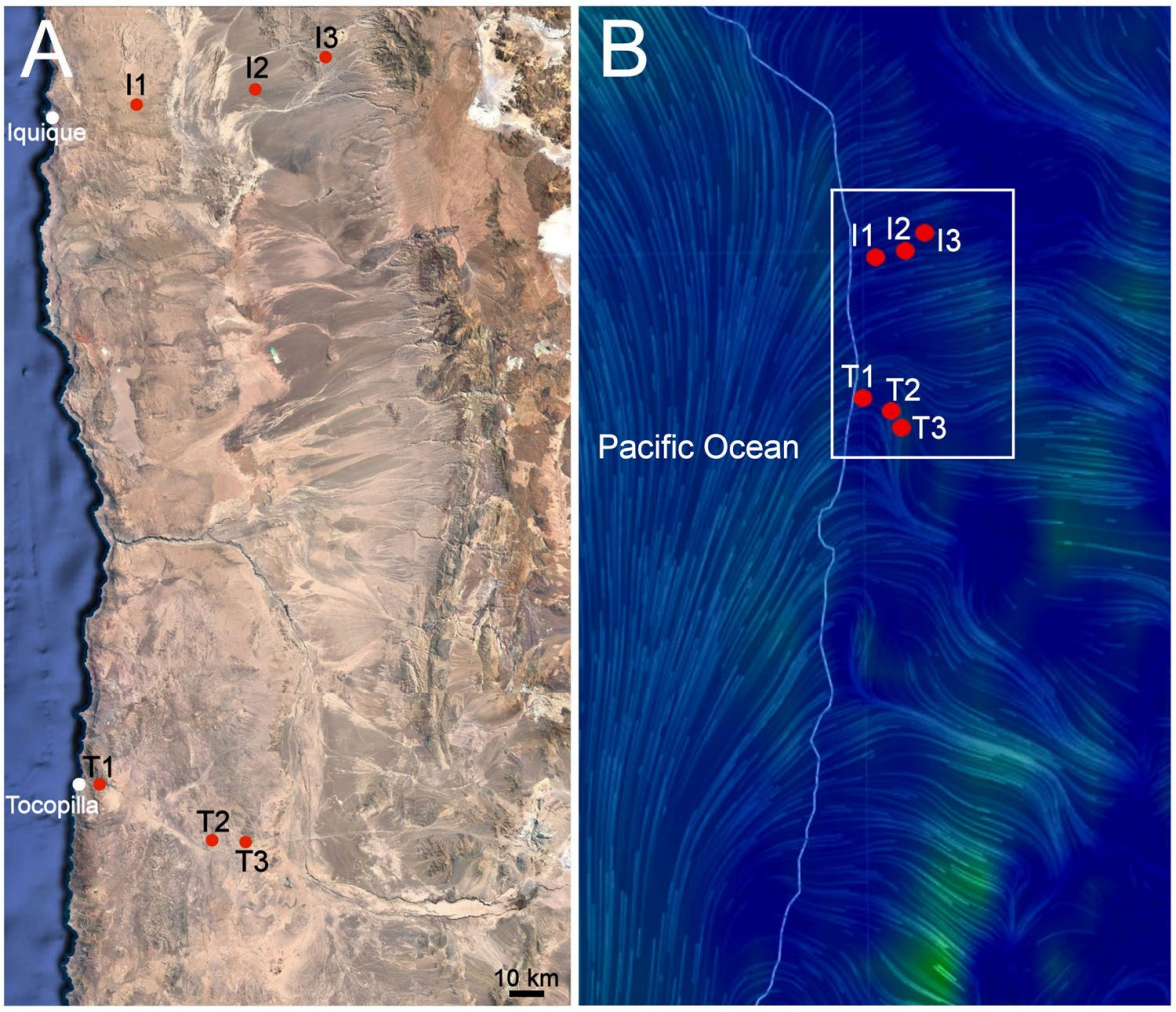
Sampling site locations and wind conditions. (**A**) Map of the Atacama Desert showing the two transects analyzed, Iquique (three sites, I1, I2 and I3) and Tocopilla (T1, T2 and T3). (**B**) Usual wind flow conditions on the two transects analyzed at 5 PM using the Earth visualization tool (https://earth.nullschool.net/about.html). Blue colors/thinner streamlines show slower winds. Greens and yellows/thicker streamlines show faster winds. The white frame in panel B depicts the zoom detailed in panel A. Note how winds flow north from the Pacific Ocean and then east into the Atacama Desert. Real time conditions may be checked at https://earth.nullschool.net/#current/wind/surface/level/orthographic=-64.85,-23.41,3000.

In order to study whether viable microbial life could use wind to move across the Atacama Desert, we devised a very simple experiment. We set two transects, each containing three sampling sites, crossing the hyperarid core of this desert (Fig. 1A) in order to follow the wind currents that in this area regularly flow north from the Pacific Ocean and then east into the hyperarid core (Fig. 1B). The first transect (the Iquique transect) was 63 km long, and the second transect (the Tocopilla transect), located 220 kms further south, was 50 km long (Fig. 1A), thus representing a total area of about 27.000 square kilometers.

To assess the identity of viable cultivable microorganisms that may be using wind transported dust to move along these two transect, five times between April and October of 2018 we set an arrays of plates containing four different growing media: ten plates of each Luria-Bertani broth, Terrific Broth, Nutrient and Marine at each site of these transects. Ten additional empty plates were also set at each site/date to quantify the amount of dust and to unveil the identity of non-cultivable microorganisms arriving at each site.

## Results

X-ray diffraction showed that the dust particles captured were composed of quartz (SiO_2_), microcline K(AlSi_3_O_8_), calcium/sodium albite (Ca)NaAlSi_3_O_8_, Clinochlore (Mg_5_Al(AlSi_3_O10)(OH)_8_), microperthite (K_0.96_Na_0.04_)AlSi_3_O_8_) and actinolite (Ca_2_Si_8_O_22_(OH)_2_) (Fig. S2), all minerals which have been reported previously on soils of the Atacama in the nearby areas of the sites sampled^7–11^.

**Figure 2:**
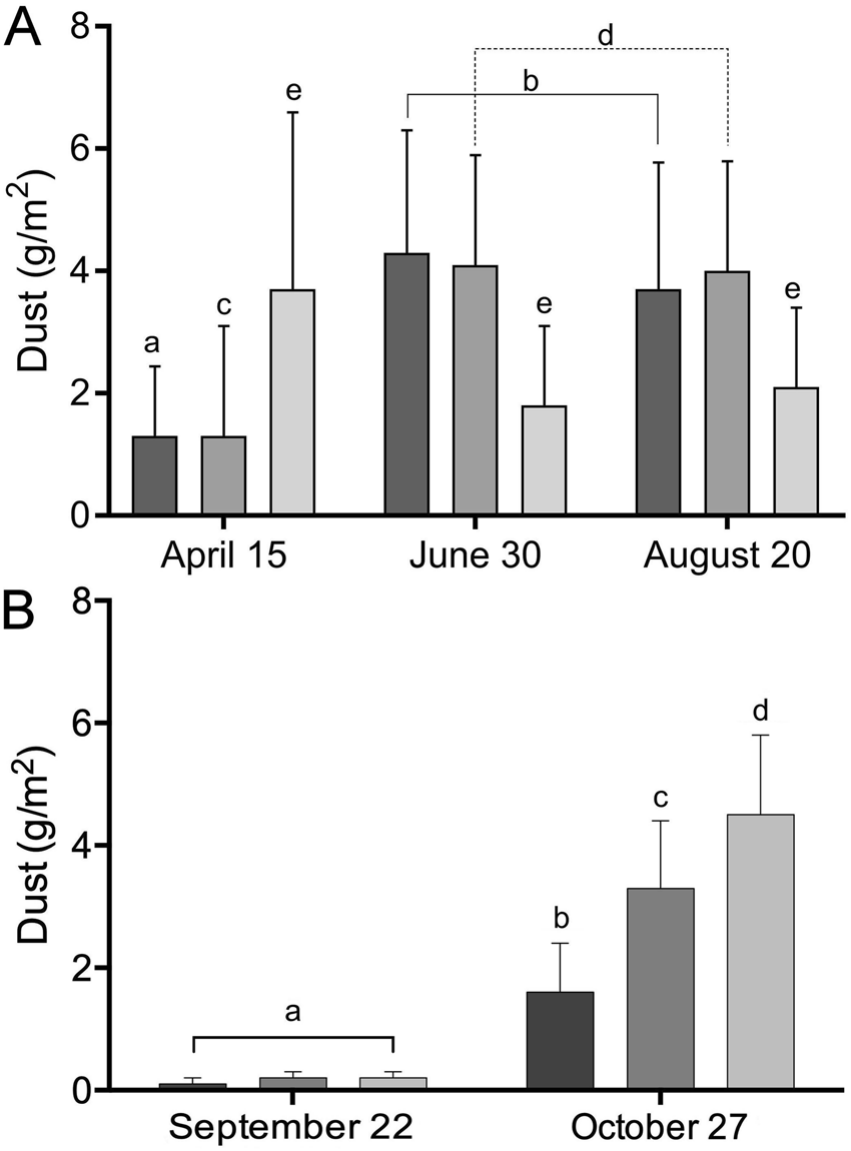
Dust captured in plates set in the hyperarid core of the Atacama Desert. (**A**) Mean dust mass captured at the sites inspected of the Iquique transect. (**B**) Mean dust mass captured at the sites inspected of the Tocopilla transect. Sites 1, 2 and 3 are represented by dark, medium and light gray bars respectively. Means with different letters are statistically different (P < 0.05; two-way ANOVA, Tukey’s post-test, see methods).

Although we observed an important intra-plate variability, we found that the amount of dust arriving at each site of the Iquique transect (Fig. 2A) was up to four times higher in the afternoon hours (June 30 and August 20 sampling dates), which is the time of the day at which winds have been reported to flow with higher speeds into the hyperarid core^12^ (Supplemental Fig. 3).

**Figure 3:**
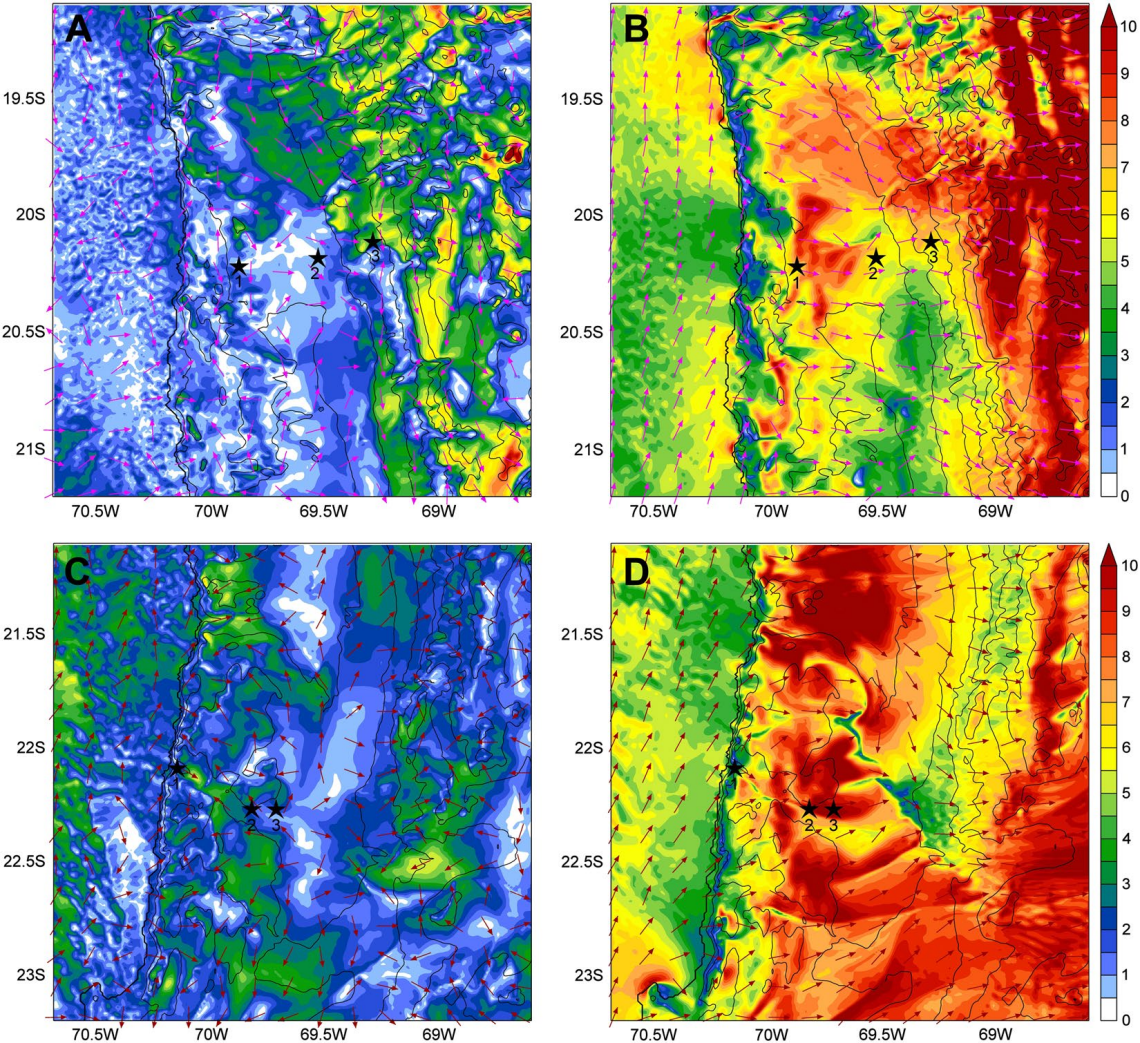
Wind vectors of the Atacama transects studied. (**A,B**), Iquique transect. In (**A**) wind vectors calculated at 10 AM of August 20 of 2018. In (**B**), wind vectors at these same sites and date, but at 5 PM. (**C,D**) Tocopilla transect. In (**C**) wind vectors calculated at 10 AM of October 27 of 2018, while in (**D**) the wind vectors at these same sites and date, but at 5 PM. In all panels arrows show the direction of the wind, and different color shading show the wind speed in ms^−1^ according to the scale show at right. Thin black lines are topographic contours, with the coast drawn as a solid black line. Black stars and numbers below show the studied sites of each transect.

**Figure 4:**
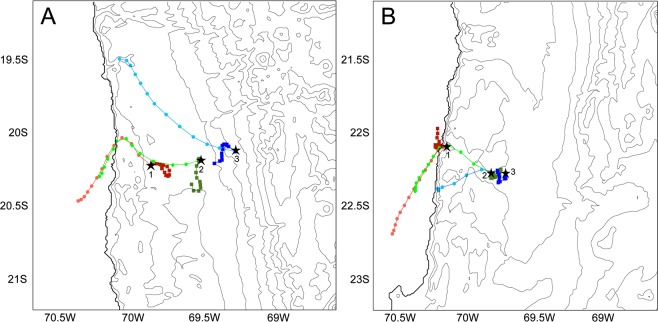
Wind speeds measured at the Tocopilla transect. Wind speeds were measured on October 27 sampling date, with means calculated from twelve measurements. Sites 1, 2 and 3 are represented by dark, medium and light gray bars respectively. Means with different letters are statistically different (P < 0.05; two-way ANOVA, Tukey’s post-test, see methods).

Atmospheric modelling confirmed the contrast in near-surface flows between morning and afternoon hours in both transects (Fig. 3). While in the morning winds at a height of 10 meters are weak and variable in direction with speeds below 4 ms^−1^ in general, (with flows that may be influenced by the features of the local landscape) (Fig. 3A,C), in the afternoon a vigorous circulation is present with wind speeds in excess of 10 ms^−1^ in some places (Fig. 3B,D). Offshore, the southerly winds are also stronger in the afternoon, while over high terrains near-surface circulation is more complex with higher wind speeds. Wind speeds measured *in situ* both in the morning (AM) and in the afternoon (PM) at the sites of the Tocopilla transect confirmed the higher speeds of the winds in the afternoon hours (Fig. 4).

**Figure 5:**
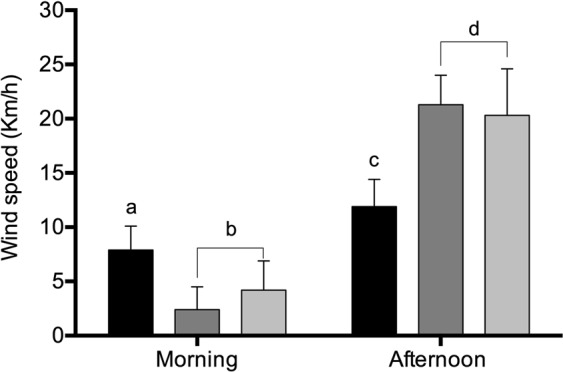
Six hours back trajectories of dust samples at the sites of the transects of the hyperarid core of the Atacama. (**A**) back trajectories calculated for the sites of the Iquique transect between 4 AM and 10 AM (dark red, dark green and dark blue squares) and between 11 AM and 5 PM (light red, light green and light blue circles) of the August 20 2018 sampling date. (**B**) back trajectories calculated for the sites of the Tocopilla transect between 4 AM and 10 AM of the October 27 2018 sampling date (dark red, dark green and dark blue squares) and between 11 AM and 5 PM (light red, light green and light blue circles). Thin black lines are topographic contours, with the coast drawn as a solid black line. Black stars and numbers below show the studied sites of each transect.

Near-surface winds snapshots of Fig. 3 allowed the calculation of morning and afternoon back trajectories for both transects shown in Fig. 5. In all cases, microbial cells collected during the morning most likely came from nearby areas (square markers in Fig. 5), while for all sites in the afternoon hours, marine aerosols and microbial life on dust particles were collected by the wind from more remote locations (circular markers in Fig. 5).

**Figure 6:**
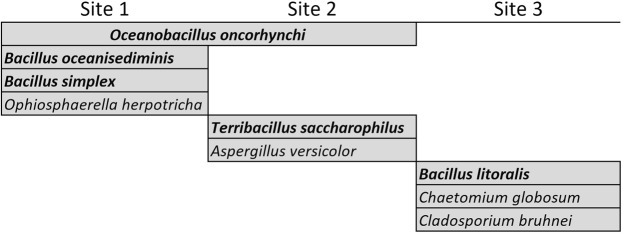
Microbial isolates identified from dust particles and its distribution at sites of the Iquique transect. Names in bold highlight bacterial species, while light font highlight fungal species.

**Figure 7:**
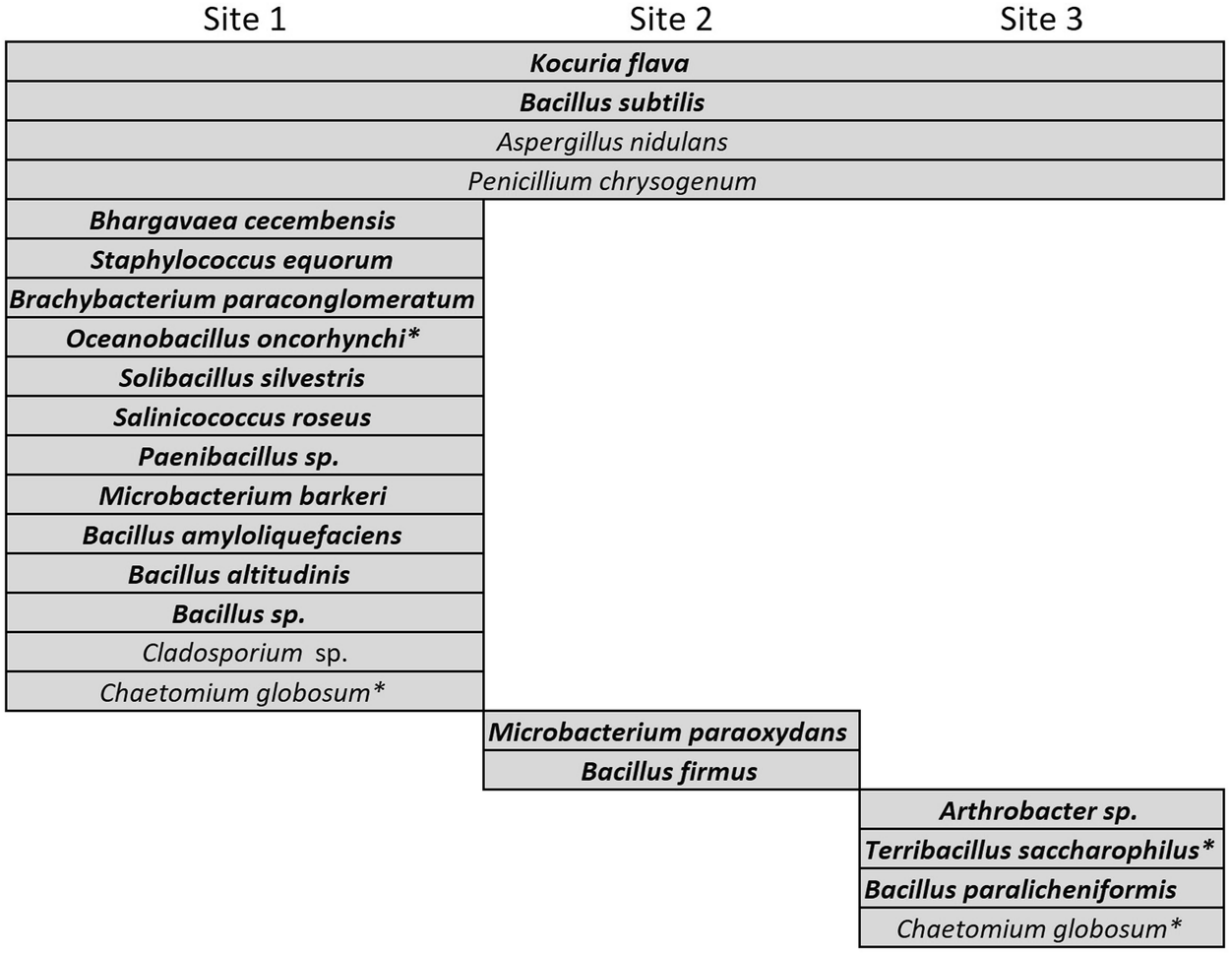
Microbial isolates identified from dust particles and its distribution at sites of the Tocopilla transect. Species names in bold highlight bacterial species, while light fonts highlight fungal species. Asterisks denote isolates also detected at the Iquique transect.

In the case of the Iquique transect, nine microbial isolates were able to be cultivated from dust particles collected at the sampling sites, (five bacterial and four fungal species) (Fig. 6). A greater diversity of species was cultivated in the Tocopilla transect (eighteen bacterial and four fungal species), of which only three were shared with the Iquique transect, thus unveiling a different airborne ecosystem (Fig. 7).

## Discussions

Our findings strongly suggest that microbial life arrives into the hyperarid core of the Atacama from the Pacific Ocean and the Coastal Range of the Atacama mainly in the afternoon hours, to be further dispersed around in the morning hours. Importantly, these findings also reveal not only the time frames in which microbial life may move across the hyperarid core of the Atacama in greater numbers, but also the time of the day at which a higher percentage of viable microorganisms may arrive, as RH increases^6,7,12^ and UV radiation decreases^12^ in the late afternoon hours at the hyperarid core, giving microbial life a higher chance to survive the transportation process. Similar to other experiments performed at the hyperarid core^6^, we detected a low percentage of colonized plates independent of the growth media used, particularly in the case of the Iquique transect. In most cases, none or only one out of ten plates exposed per site/sampling date showed signs of microbial growth after two weeks of observation after inoculation (Table S1). Despite the limitations of the culture-dependent approach, this allowed us to detect a number of new microbial isolates in both transects.

The species detected at site 1 of the Iquique transect suggested the potential source of origin and subsequent transport route of microbial life through the hyperarid core; *Oceanobacillus oncorhynchi* is a halotolerant obligate alkaliphile first described in watery environments^13^, which in this case may had arose from either the Pacific Ocean and/or from the coast of the Atacama (the first area of this desert encountered by the winds coming from the ocean and then moving into the hyperarid core) explaining why it is detected at the sites closer to the coast in both transects. *Oceanobacillus* species were first isolated from deep-sea sediments lending support to this hypothesis^14^, an observation which also applies to *Bacillus oceanisediminis*, first isolated from marine sediments from the South Sea in China^15^. In turn, bacterial species like *Bacillus simplex* have been isolated from the plant rhizosphere^16^, suggesting a potential origin from the soils of the top of the Coastal Range of the Atacama, which contain a few plant species that use fog as their main source of water^17^, plants which are completely absent in the coast of the Atacama and also further inland in the inspected region. A similar case applies to *Ophiosphaerella herpotricha*, a fungal species known to affect the roots of grass species^18^.

An analogous analysis applies to the species detected further inland (sites 2 and 3); *Terribacillus saccharophilus* is a moderately halophilic bacterial species isolated from soils in Japan and is closely related to species of the *Oceanobacillus* genus^19^, and may also have originated at the coast of the Atacama and then moved further inland. This could also be the case of *Bacillus litoralis*, an halophilic bacterial species isolated from a tidal flat of the Yellow Sea in Korea^20^, while *Cladosporium bruhnei* belongs to a genus with several species isolated from hypersaline environments^21^. Other species again suggest an origin at the Coastal Range hills; *Aspergillus versicolor* is a highly ubiquitous fungus commonly isolated from soil, plant debris and marine environments^22^, and *Chaetomium globosum* is a mesophilic saprophytic fungus that primarily resides in habitats like mountain soils across various biomes^23^.

Similar to the Iquique transect, a number of bacterial species in the sites closer to the coast of the Tocopilla transect also suggest a marine origin; *Oceanobacillus oncorhynchi*, *Bhargavaea cecembensis* (isolated from deep-sea sediments samples collected at the Chagos-Laccadive ridge system in the Indian Ocean^24^), and *Brachybacterium paraconglomeratum*, which belongs to a small genus containing species isolated from coastal sands^25^. In turn, *Salinicoccus roseus* is a moderately halophilic bacterium isolated from solar salterns in Spain^26^, and *Microbacterium barkeri* is a moderately halophilic actinobacteria first isolated from sea-water samples taken from Amursky Bay of the Gulf of Peter the Great (Russia)^27^.

In turn, other species may have also arose from the sparse plant-covered areas of fog oases on top of the hills of the Coastal Range; *Solibacillus silvestris* is a moderately halophilic bacterium first found in forest soils^28^, members of the genus *Paenibacillus* are facultatively anaerobic bacteria and have been isolated from decomposing plant material^29^, *Bacillus amyloliquefaciens* is a Gram-positive found as part of the plant rhizosphere^30,31^, *Microbacterium paraoxydans* has been isolated from arsenic polluted vegetated soils^32^, *Bacillus firmus* is an alkaliphilic bacterium that has been isolated from oil reservoirs and vegetation covered soils^33^, while *Bacillus paralicheniformis* has been isolated from soybean and other plants^34,35^ (the case of *Bacillus firmus* is of particular interest, because although it was found at site 2, is one of the few microbial species able to survive in María Elena^6^, the driest place on the Atacama and on Earth, located only eleven kilometers further east from site 2). A higher number of plant-associated microorganisms along the Tocopilla transect may be explained by the fact that the amount and diversity of plants at the Coastal Range hills steadily increases south.

Interestingly, two of the species detected by us have been reported as airborne bacteria; *Kocuria flava*^36^, an actinobacteria isolated in Xinjiang, China, and *Bacillus altitudinis*, collected in the atmosphere at altitudes between 24 and 41 km during a balloon flight from Hyderabad, India^37^. Whether these two species may have arrived from even more distant places is been now investigated.

**Figure 8:**
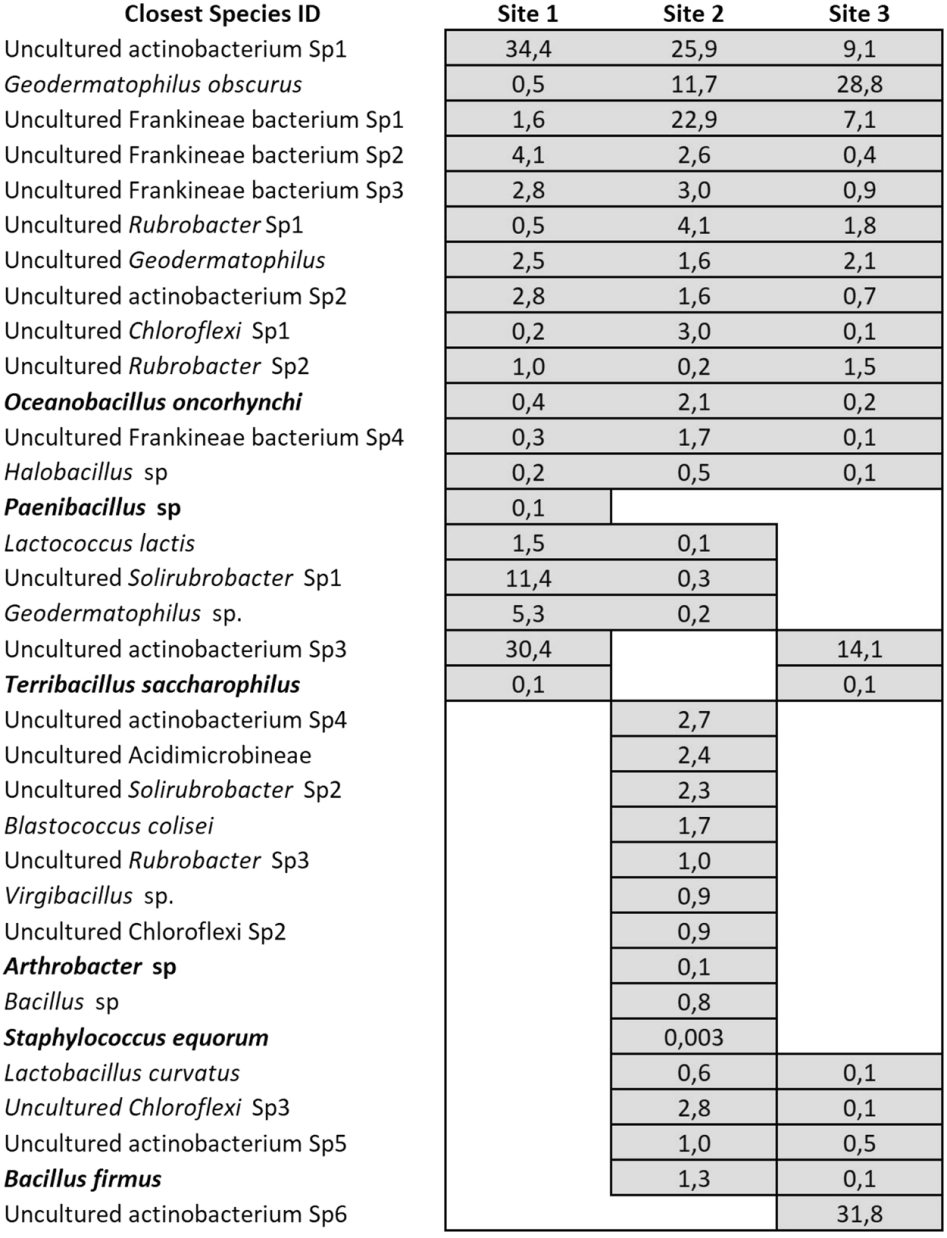
Main OTUs identified from DNA extracted from dust particles and its distribution at the sites of the Tocopilla transect. Species names in bold highlight bacterial species also detected by cultivation. Values inside site distribution bars indicate percentages of total OTUs.

Direct DNA extraction from dust particles unveiled a number of bacterial species already detected by cultivation (*Oceanobacillus oncorhynchi*, *Paenibacillus* sp, *Terribacillus saccharophilus*, *Arthrobacter* sp., *Staphylococcus equorum*, and *Bacillus firmus*), (Fig. 8), but also a greater number of bacterial species not detected by the culture dependent method used. Although archaea specific primers were used, no archaeal sequences were found. This is puzzling, and may be explained by the absence of sources of origin for these type of species in the transects analyzed. If one of our transects had crossed the Salar Grande located at the Coastal Range of the Atacama (where archaea has been reported^38^), we may have detected them, provided that they are as resilient to wind transport as shown for bacteria and fungi.

Similar to the analysis shown above, the microbial species detected by direct DNA extraction from dust particles again suggest the source of origin for the species found inland; *i.e*., *Oceanobacillus oncorhynchi* and *Halobacillus* (Pacific Ocean/Coastal Range) or the Coastal Range of the Atacama (*Lactobacillus curvatus*, *Bacillus firmus*).

Several of the species found by both culture dependent and independent methods used have been reported in the inspected region; *i.e*., *Bacillus simplex*^39,40^, *Bacillus litoralis*^41^, *Bacillus subtilis*^42^, *Aspergillus nidulans*^43^, *Penicillium chrysogenum*^44^, *Bacillus amyloliquefaciens*^45^, and *Geodermatophilus obscurus*^6^.

It is also interesting that 71% of the microbial species detected either by cultivation or direct DNA extraction from the dust samples collected can also use spores to disperse in the environment (*e.g*., *Bacillus* and fungal species), suggesting a fraction of the species that may also be arriving as spores, with the remaining 29% of the species (*e.g., Microbacter, Brachybacterium*) that should be using only wind-transported dust to reach the hyperarid core of the Atacama.

Altogether, the analysis of dust particles collected across the hyperarid core of the Atacama shows that microbial life is able to efficiently move across the driest and most UV irradiated desert on Earth unharmed, particularly in the late afternoon hours. Considering the wind speeds measured in the studied transects (~10–20 km/h), and the distances covered (~100 km), the total transport time required by microbial life to reach deep into the hyperarid core of the Atacama from their source origin is only 5–10 hours, transport times that will vary depending on the season of the year. Thus, the result presented here are consistent with the hypothesis that the microbes detected in the soils of the hyper-arid core of the Atacama - delineated by the presence of nitrate deposits - are carried there by the wind with transport times of days or less. Given the aforementioned explanations, and that the transects studied are separated by more than 200 kms (a distance selected with the purpose of understanding whether these are processes that extend to all of the Atacama), we may assume with confidence that our conclusions apply to the other regions of this desert, that is, that the main driver for the transport of microbial life in this desert is the presence and speed of winds.

Our data unveiled the potential point of origin for the species found at the hyperarid core of the Atacama in the coasts of this desert and its Coastal Range. What happens to these species after arriving to the hyperarid core is now been investigated, as well as whether the atmospheric transport of microbial life and subsequent arrival may be affected by the recent unusual rain events in this region^45^.

### Implications for mars

It is well known that Mars is constantly affected by winds^46^ and dust storms that in many cases can cover its entire surface^47–49^. Our results suggest that any potential viable microbial life on Mars may similarly spread all over significant distances using dust-mediated transport, either now or in the past, and that extreme aridity does not fully prevent this from taking place.

The Martian atmosphere is full of  dust, with loads that greatly fluctuate with the season of the year^50^. Airborne dust interacts with solar visible and thermal infrared radiation, perturbing atmospheric heating, modifying the circulation and thermal structure of the atmosphere, and ultimately leading to sudden atmospheric perturbations which cause the uplift of dust (thus potentially collecting new microbial cells from different regions of the planet) and the subsequent development of planet-encircling sandstorms^48,49^ (which then may redistribute these microbes). Therefore, it would be interesting to collect and analyze fresh samples of airborne Martian dust in search of potential biosignatures.

The potential transportation of microbes by Martian dust would have been (or may be) of particular relevance in a planet where habitable niches may have been separated both in time and space^51^, particularly after the Noachian period^52^. Favorable conditions for the appearance and continuity of life on Mars may have been met at different times and discrete places, spanning a minimum time range of about 2 million years^53^. As it has been argued that the lack of continuously hospitable conditions would have deterred a continuous biological evolution on Mars^53^, our results offer a way to sort out this problem, as the lack of inter-connectivity between dispersed habitable environments by ancient water flow or tectonics may have been balanced by wind dispersal of microorganisms during extended periods of time. As a consequence, the aeolian distribution of life may have allowed some degree of evolution of microbial Martian life forms.

Finally, our results are of critical application for planetary protection, as terrestrial microorganisms hitchhiking in rovers and landers (and their discarded landing material) may have been widely dispersed all over the Martian surface by planetary-level dust storms^54^.

## Materials and Methods

### Sampling sites and dates

The Iquique transect sampling coordinates were: Site 1; 20°13′1.81″S, 69°52′57.70″W, elevation 1106 m. Site 2; 20°10′53.30″S, 69°32′6.90″W, elevation 1129 m. Site 3; 20°6′46.71″S, 69°17′40.70″W, elevation 2300 m. Samples were taken on April 15, June 30 and August 20 of 2018. The inter-site distances were: Coast-Site1: 28 km, Site1-Site 2: 36 km, Site 2-Site 3: 27 km.

The Tocopilla transect sampling coordinates were: Site 1; 22°5′13.72″S, 70° 9′22.90″W, elevation 440 m. Site 2; 22°16′0.72″S, 69°49′44.60″W, elevation 1450. Site 3; 22°16′4.63″S, 69°43″19.31″W, elevation, 1304. Samples were taken on September 22 and October 27 of 2018. Inter-site distances were: Coast-Site1: 4 km, Site1-Site2: 39 km, Site2-Site3: 11 km.

### X-Ray diffraction

Samples, ground into powders with an agate mortar and pestle (Pulverisette 2, Fristsch) and X-ray powder diffraction data were collected using a Bruker D8 Eco Advance in Bragg–Brentano geometry, Cu Kα radiation and Lynxeye XE-T linear detector. The X-ray generator was operated at 40 KV and 25 mA. Samples were scanned in step of 0.05° (2θ), over of range 5°–60° (2θ) with collection time of 1 s at each point. The phase identification was performed by comparing the measured diffraction pattern (diffractograms) with patterns of the PDF Database with the DIFFRAC.EVA software (Bruker AXS).

### Wind vectors and back trajectory plots

The 10-meter horizontal wind vectors and the back-trajectory plots are generated using the output data from a Weather Research and Forecasting (WRF) model simulation over the Atacama Desert for each period of interest. Back trajectories are computed using the Hybrid Single-Particle Lagrangian Integrated Trajectory model^55^ (HYSPLIT) forced offline with the WRF output, developed by the National Oceanic and Atmospheric Administration’s Air Resources Laboratory. HYSPLIT linearly interpolates in space and time the WRF raw output data and computes back trajectories by following individual air parcels (i.e. Lagrangian framework).

WRF version 3.9.1.1 is forced with the 6-hourly data of the National Centers for Environmental Prediction Climate Forecast System Reanalysis^56^, spatial resolution of 0.5° × 0.5°, for the periods 30th June–6th July, 15th August–23rd August, 21st–22nd September and 26th–27th October 2018. The model is run in a 4-nested configuration with the outermost grid at a spatial resolution of 27 km and the inner nests at 9 km, 3 km and 1 km. The 10-minute output of the 1 km grid is post-processed and subsequently used for analysis. In the vertical 60 levels are considered, concentrated in the planetary boundary layer, with the model top at 30 hPa. Further details about the model configuration are given in Fonseca *et al*.^57^.

### Wind flow maps

The wind flow maps shown in panel B of Figs 1 and S1 were modified from the wind flow maps available at the earth.nullschool.net web page, which images are generated by the Global Forecast System (GFS), a product of NCEP of the National Weather Service, NOAA.

### Cultivation of isolates from dust particles

Sterile Petri dishes containing agar and four different growing media (ten of each Luria-Bertani Broth, Terrific Broth, Nutrient agar and Marine Media) were left for one hour at the inspected sites in order to trap dust particles, aseptically sealed and growth observed for 2 weeks. Colonies were then separated and subcultured in order to obtain enough biomass for DNA extraction. In addition, empty Petri dish plates were left (10 per sampling site at each sampling date) to assess the amount of dust arriving at each site.

### Illumina NGS-Based 16S rRNA and 18S rRNA sequencing

DNA extraction: 10 sterile Petri dishes and four sterile plastic boxes were used to aseptically collect dust for one hour during the October 22 sampling date at the Tocopilla transect. DNA was extracted from these pellets using the DNeasy PowerSoil Kit according the manufacturer instructions, except that at the cell lysis step, two pulses of 2 minutes were used in a FastPrep-24 5G homogenizer (MP Biomedicals). Three ng of purified DNA were then amplified in a first PCR of 26 cycles for 16S, and 29 cycles for archaea and 18S with Q5 Hot Start High-Fidelity DNA Polymerase (New England Biolabs) in the presence of 100 nM primers (5′ACACTGACGACATGGTTCTACACCTACGGGNGGCWGCAG3′ and 5′

TACGGTAGCAGAGACTTGGTCTGACTACHVGGGTATCTAATCC3′) for V3-V4 16S rDNA, 200 nMprimers (Arch1F-CS1 5′ACACTGACGACATGGTTCTACACGGRAAACTGGGGATAAT3′ and Arch1R-CS2 5′TACGGTAGCAGAGACTTGGTCTTRTTACCGCGGCGGCTGBCA3′) for archaea and 200 nM primers (563f-CS1 5′ACACTGACGACATGGTTCTACAGCCAGCAVCYGCGGTAAY3′ and 1132R-CS2 5′TACGGTAGCAGAGACTTGGTCTCCGTCAATTHCTTYAART3′) for 18S rDNA. After the first PCR, a second PCR of 15 cycles was perfomed with Q5® Hot Start High-Fidelity DNA Polymerase (New England Biolabs) in the presence of 400 nM of primers (5′AATGATACGGCGACCACCGAGATCTACACTGACGACATGGTTCTACA-3′ and 5′-CAAGCAGAAGACGGCATACGAGAT- [10 nucleotides barcode]-TACGGTAGCAGAGACTTGGTCT-3′) of the Access Array Barcode Library for Illumina Sequencers (Fluidigm). The obtained amplicons were validated and quantified by a Bioanalyzer, and equimolecular pools were purified using AMPure beads or agarose gel and titrated by quantitative PCR using the “Kapa-SYBR FAST qPCR kit for LightCycler480” and a reference standard for quantification. The pool of amplicons was denatured prior to be seeded on a flowcell at a density of 10pM, where clusters were formed and sequenced using a “Miseq Reagent kit v3” in a 2 × 300 sequencing run on a MiSeq sequencer”.

Raw sequences from bacterial 16S rRNA and eukaryotic 18S rRNA regions were processed in MOTHUR software v.1.40.5^58^, using a custom script based upon MiSeq SOP^59^. Briefly, reads containing below a minimum number of bp (≤440 bp for Bacteria), those containing ambiguous nucleotide identities (‘Ns’) and/or homopolymers longer than 8 bp, as well as singletons and/or those identified as putatively chimeric, were removed from subsequent analyses. Remaining sequence reads were then clustered into OTUs (Operational Taxonomic Units) at the 97% similarity level. Datasets were finally rarefied independently to even sequencing depth, corresponding to the lesser number of sequences found in the samples (86847 for Bacteria). Taxonomic affinities for bacterial reads were assigned by comparison of OTUs representative sequences against RDP database (RDP v.16 reference files; release 11. Sequences assigned to ‘mitochondria’ or chloroplast were removed from further analyses. Only OTUs with more than 500 sequences were shown in tables 1 and 2.

### DNA extraction from isolates

DNA was extracted as detailed for Illumina NGS-Based 16S rRNA Sequencing.

### 16S and 18S rRNA amplification and sequencing from isolates DNA

16S rRNA of bacterial isolates was amplified using the GoTaq Green Master Mix (Promega) and the primers 341 f (5′CCTACGGGNGGCWGCAG3′) and 785r (5′GACTACHVGGGTATCTAATCC3′). PCR conditions used were: 95 °C for 5 min, and 25 cycles of (95 °C for 40 s, 55 °C for 2 min, 72 °C for 1 min) followed by 72 °C for 7 min. The resultant reactions were visualized in a 2% agarose TAE gel ran at 50 V for 40 min.

18S rRNA of fungal isolates was isolates was amplified using the GoTaq Green Master Mix (Promega) and the primers 566 f (5′CAGCAGCCGCGGTAATTCC3′) and 1200r (5′CCCGTGTTGAGTCAAATTAAGC3′). PCR conditions used were: 95° C for 15 min, and 30 cycles of (95 °C for 45 s, 55 °C for 1 min, 72 °C for 2.5 min) followed by 72 °C for 7 min. The resultant reactions were visualized in a 2% agarose TAE gel at 50 V.

The automated sequencing of the resulting PCR products was conducted by Macrogen DNA Sequencing Inc. (Seoul, Korea). Sequences were checked for quality using the BioEdit software (Ibis Therapeutics) and end-trimmed before using the Megablast option for highly similar sequences of the BLASTN algorithm against the National Centre for Biotechnology Information nonredundant database (www.ncbi.nlm.nih.gov) to search for the closest species of each of the isolates obtained. Only species with at least 98% of sequence identify and an E value of 0.0 were selected, and only species with defined genus and species names were considered for phylogenetic closeness.

### Wind speed

Wind speed was measured with a portable Smart Sensor Electronic Anemometer (model AR816). Twelve measurements per site were taken.

### Statistical methods

The statistical test used was a two-way ANOVA and for multiple comparisons, a Tukey *a posteriori* test. A two-tail was selected because our null hypothesis (there is no interaction between geographical variation (columns) and temporal variations (rows)) for all experiments and measurements could be rejected by a difference either a positive or negative direction. The alpha selected was 0,05 (5%). The error bars represent the standard deviation (SD). Measurements were repeated ten times for dust capture experiments and twelve times for wind speed measurements. Replicas in our study represent the number of plates randomly set in each site to avoid pseudoreplication. Further details on statistic can be seen in the Supplementary Information File.

## Supplementary information

Supplementary Information

## Data Availability

The authors declare that all the data supporting the findings of this study are available within the article (and its Supplementary Information file), or available from the corresponding authors on reasonable request.
