# Author Correction: Aeolian transport of viable microbial life across the Atacama Desert, Chile: Implications for Mars

**DOI:** 10.1038/s41598-020-57444-6

**Published:** 2020-01-15

**Authors:** Armando Azua-Bustos, Carlos González-Silva, Miguel Ángel Fernández-Martínez, Cristián Arenas-Fajardo, Ricardo Fonseca, F. Javier Martín-Torres, Maite Fernández-Sampedro, Alberto G. Fairén, María-Paz Zorzano

**Affiliations:** 1 Centro de Astrobiología (CSIC-INTA), 28850 Madrid, Spain; 2 Instituto de Ciencias Biomédicas, Facultad de Ciencias de la Salud, Universidad Autónoma de Chile, Santiago, Chile; 3 Facultad de Ciencias, Universidad de Tarapacá, Arica, Chile; 4 Atacama Biotech, Santiago, Chile; 5 Division of Space Technology, Department of Computer Science, Electrical and Space Engineering, Luleå University of Technology, Luleå, Sweden; 6 Instituto Andaluz de Ciencias de la Tierra (UGR-CSIC), Armilla, Granada Spain; 7 Department of Astronomy, Cornell University, Ithaca, 14853 NY USA

Correction to: *Scientific Reports* 10.1038/s41598-019-47394-z, published online 22 August 2019

This Article contains an error in the order of the Figures. Figures 4 and 5 were published as Figures 5 and 4 respectively. The correct Figures 4 and 5 appear below as Figures 1 and 2.

**Figure 1:**
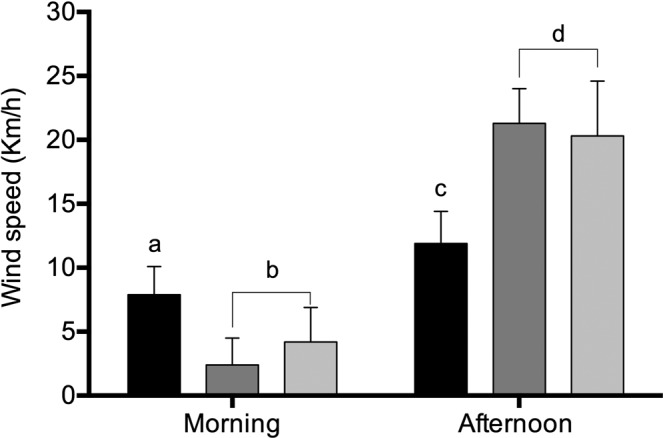
.

**Figure 2:**
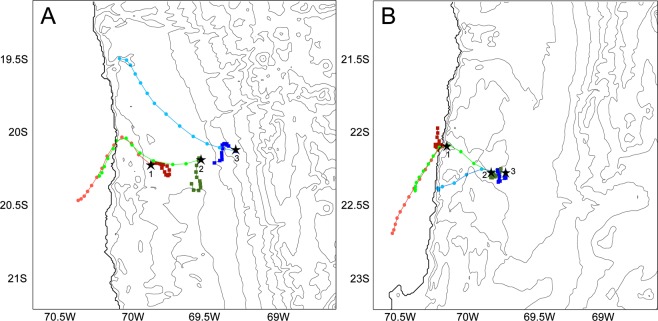
.

