# Supplementary material for: Aeolian transport of viable microbial life across the Atacama Desert, Chile: Implications for Mars
Source: Sci Rep. 2019 Aug 22;9:11024. doi: 10.1038/s41598-019-47394-z (PMC6706390; doi:10.1038/s41598-019-47394-z)
Supplement: Supplementary file 1 — Supplementary Information [file 41598_2019_47394_MOESM1_ESM.pdf]

Supplementary Information

Aeolian transport of viable microbial life across the Atacama Desert, Chile: Implications for Mars

Armando Azua-Bustos, Carlos González-Silva, Miguel Ángel Fernández-Martínez, Cristián Arenas-Fajardo, Ricardo Fonseca, F. Javier Martín-Torres, Maite Fernández-Sampedro, Alberto G. Fairén, María-Paz Zorzano

Supplemental Tables and Figures

Supplemental Table S1.- Plate colonization by growing media in the Iquique and Tocopilla transects. Marine; marine media, LB; Luria-Bertani broth, TB; Terrific broth. A total of 10 plates per media were used.

	Iquique Transect				Tocopilla Transect		
	Marine	LB	TB		Marine	LB	TB
**April 15**				**Sept 22**			
I1	1	1	2	T1	2	4	6
I2	0	1	2	T2	1	2	2
I3	0	1	1	T3	0	2	4
**June 30**				**Oct 27**			
I1	1	0	3	T1	1	1	1
I2	0	1	1	T2	2	0	1
I3	1	0	0	T3	1	1	1
**August 20**							
I1	4	0	3				
I2	0	3	2				
I3	0	3	2				

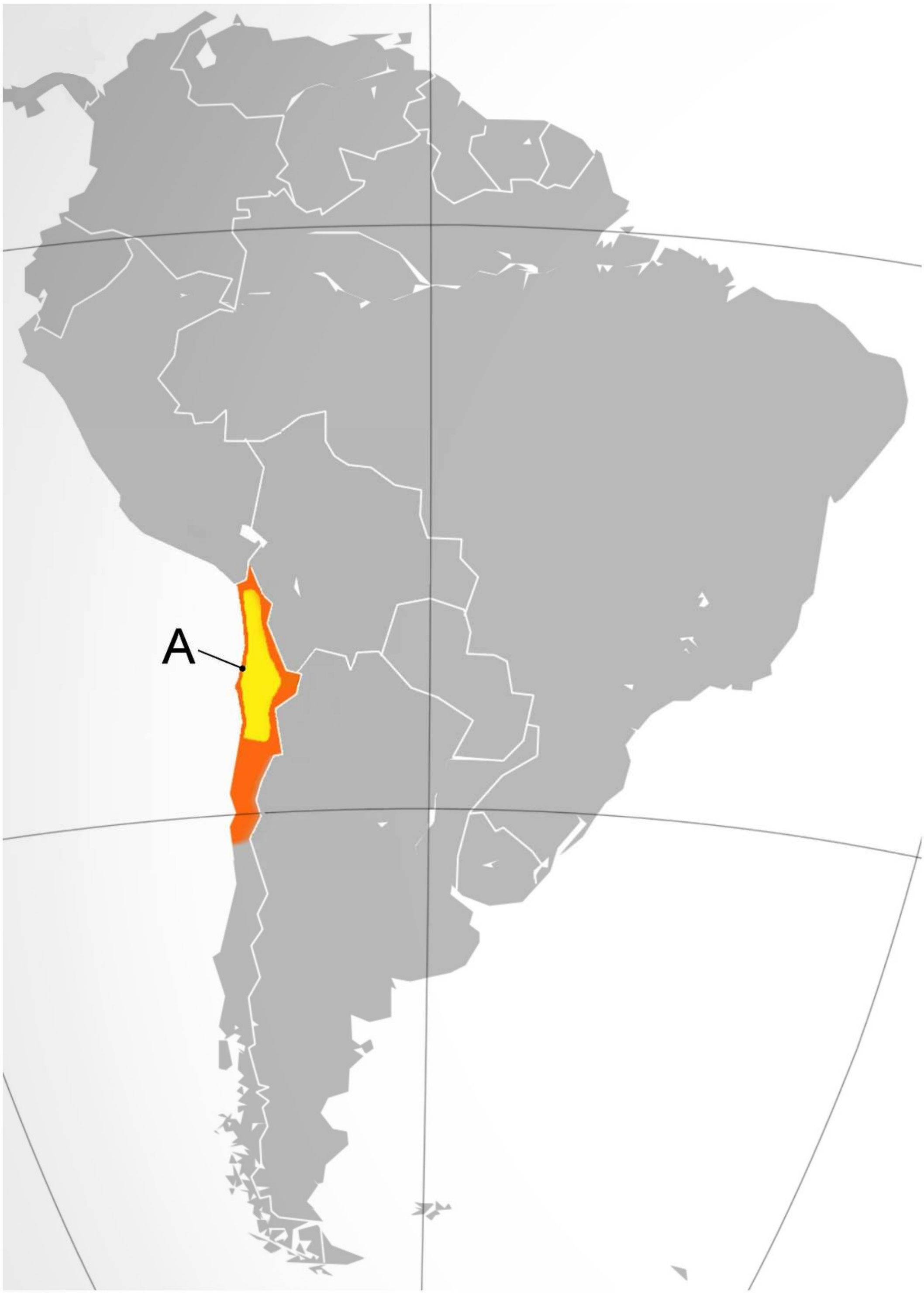

Supplemental Figure S1.- Location of the Atacama Desert in South America. In orange, the Atacama Desert. In yellow, the hyperarid core of the Atacama. A, Coastal Range of the Atacama Desert.

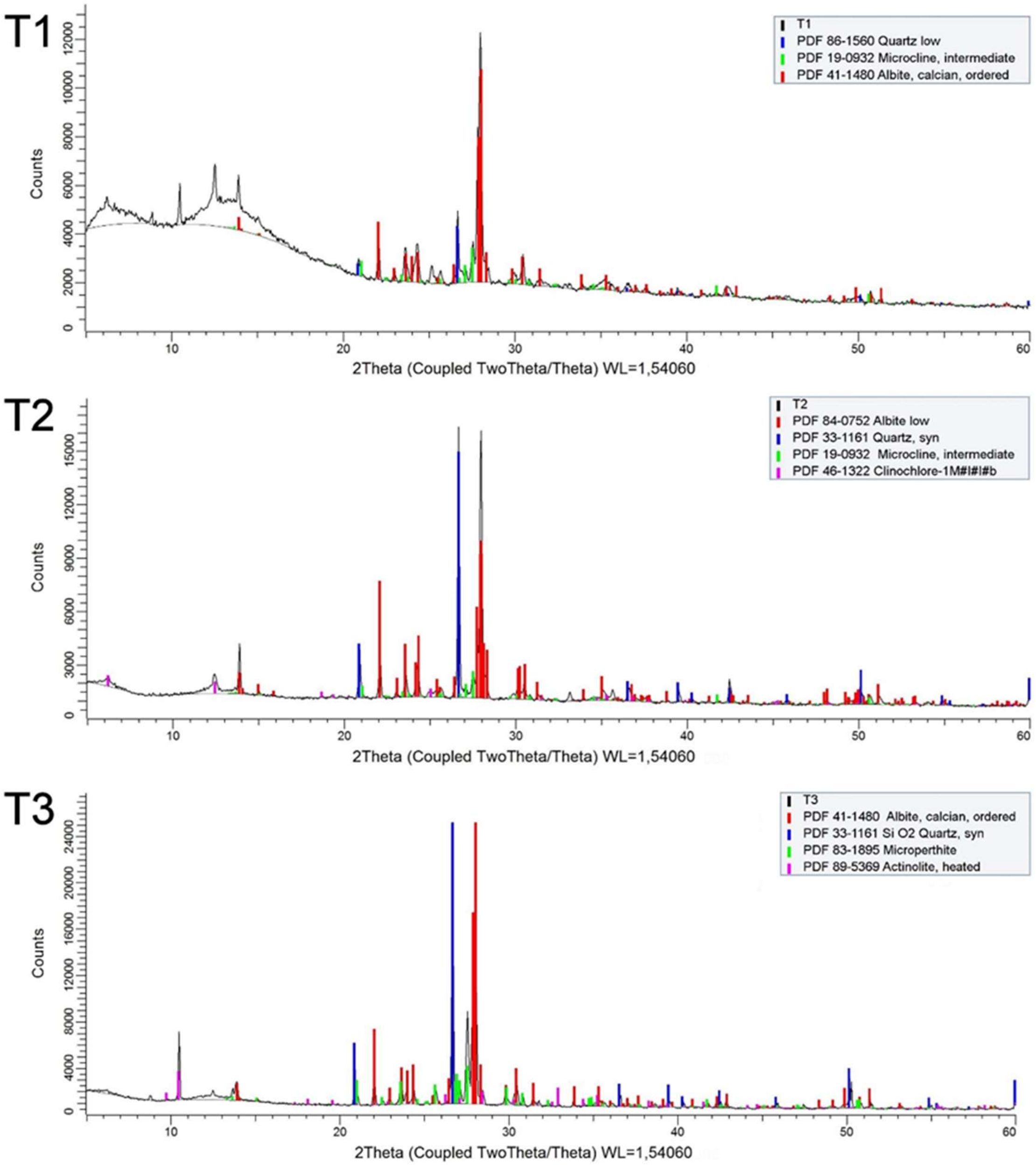

Supplemental Figure S2.- XRD graphs of the sampled dust particles. T1, T2 and T3 are the sampled sites of the Tocopilla transect.

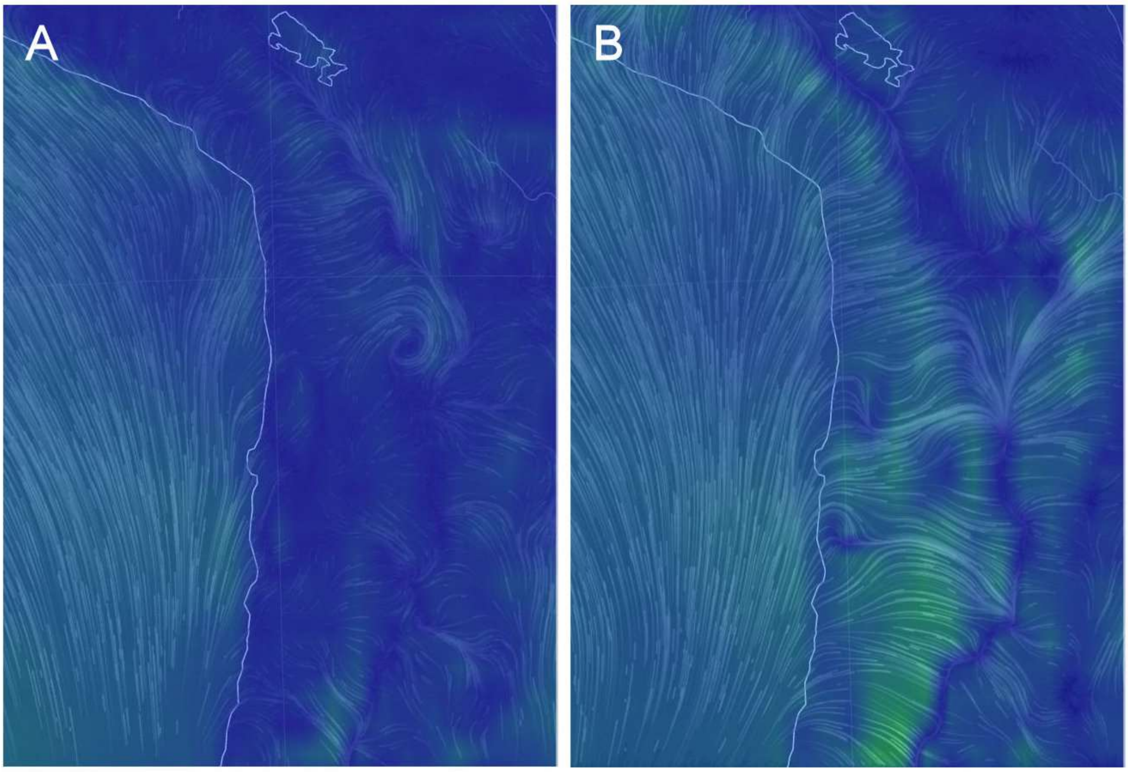

Supplemental Figure S3.- Wind speed as determined with the Earth visualization tool (https://earth.nullschool.net/about.html). A) Wind map of the Atacama Desert focused in the studied region as seen at 10 AM. B) Same region as seen in A but at 5 PM of October 27 of 2018. Blue colors/thinner streamlines show slower winds. Greens and yellows/thicker streamlines show faster winds.

Statistical Methods

The statistical test used was a two-way ANOVA and for multiple comparisons, a Tukey *a posteriori* test. A two-tail was selected because our null hypothesis (there is no interaction between geographical variation (columns) and temporal variations (rows)) for all experiments and measurements could be rejected by a difference either a positive or negative direction. The alpha selected was 0,05 (5%). The error bars represent the standard deviation (SD). Measurements were repeated ten times for dust capture experiments and twelve times for wind speed measurements. Replicas in our study represent the number of plates randomly set in each site to avoid pseudoreplication.

A) Dust captured experiments

Interactions between row and column show differences between rows that are not the same at each column, equivalent to the variation between columns that are not the same at each row. The Row factor correspond to temporal variation (different months) and column factor represent geographical sites of sampling across of transects (site 1, site 2 and site 3 respectively). The residual or error is the vriation among replicates not related to systematic differences between rows and columns.

SS = Sum of squares; DF = degrees of freedom; MS = mean square; F = F ratio.

Iquique transect (n=90)

ANOVA table	SS	DF	MS	F (DFn, DFd)	P value
Interaction	91.04	4	22.76	F (4, 81) = 5.081	P = 0.0011
Row Factor	30.69	2	15.34	F (2, 81) = 3.425	P = 0.0373
Column Factor	6.822	2	3.411	F (2, 81) = 0.7614	P = 0.4703
Residual	362.9	81	4.48		

Tocopilla transect (n=60)

ANOVA table	SS	DF	MS	F (DFn, DFd)	P value
Interaction	19.73	2	9.867	F (2, 54) = 16.58	P < 0.0001
Row Factor	132.0	1	132.0	F (1, 54) = 221.9	P < 0.0001
Column Factor	22.80	2	11.40	F (2, 54) = 19.16	P < 0.0001
Residual	32.13	54	0.5950		

B) Wind speed measurements (n=72)

In this table the row factor correspond to temporal variations (morning and afternoon) and the column factor represent the geographical sites of sampling across transects (site 1, site 2 and site 3 respectively).

ANOVA figure	SS	DF	MS	F (DFn, DFd)	P value
Interaction	752.5	2	376.3	F (2, 66) = 46.48	P < 0.0001
Row Factor	75.88	2	37.94	F (2, 66) = 4.687	P = 0.0125
Column Factor	3042	1	3042	F (1, 66) = 375.8	P < 0.0001
